# Suppression of PI3K/Akt/mTOR/c-Myc/mtp53 Positive Feedback Loop Induces Cell Cycle Arrest by Dual PI3K/mTOR Inhibitor PQR309 in Endometrial Cancer Cell Lines

**DOI:** 10.3390/cells10112916

**Published:** 2021-10-27

**Authors:** I-Lun Hsin, Huang-Pin Shen, Hui-Yi Chang, Jiunn-Liang Ko, Po-Hui Wang

**Affiliations:** 1 Institute of Medicine, Chung Shan Medical University, Taichung 40201, Taiwan; zontetsuken@gmail.com (I.-L.H.); 52carboplatin@gmail.com (H.-P.S.); mskee53@gmail.com (H.-Y.C.); jlko@csmu.edu.tw (J.-L.K.); 2 Department of Obstetrics and Gynecology, Chung Shan Medical University Hospital, Taichung 40201, Taiwan; 3 Department of Medical Research, Chung Shan Medical University Hospital, Taichung 40201, Taiwan; 4 School of Medicine, Chung Shan Medical University, Taichung 40201, Taiwan; 5 Division of Medical Oncology, Department of Internal Medicine, Chung Shan Medical University Hospital, Taichung 40201, Taiwan

**Keywords:** c-Myc, endometrial cancer, mutant p53, dual PI3K/mTOR inhibitor, PQR309

## Abstract

Gene mutations in PIK3CA, PIK3R1, KRAS, PTEN, and PPP2R1A commonly detected in type I endometrial cancer lead to PI3K/Akt/mTOR pathway activation. Bimiralisib (PQR309), an orally bioavailable selective dual inhibitor of PI3K and mTOR, has been studied in preclinical models and clinical trials. The aim of this study is to evaluate the anticancer effect of PQR309 on endometrial cancer cells. PQR309 decreased cell viability in two-dimensional and three-dimensional cell culture models. PQR309 induced G1 cell cycle arrest and little cell death in endometrial cancer cell lines. It decreased CDK6 expression and increased p27 expression. Using the Proteome Profiler Human XL Oncology Array and Western blot assay, the dual inhibitor could inhibit the expressions of c-Myc and mtp53. KJ-Pyr-9, a c-Myc inhibitor, was used to prove the role of c-Myc in endometrial cancer survival and regulating the expression of mtp53. Knockdown of mtp53 lowered cell proliferation, Akt/mTOR pathway activity, and the expressions of c-Myc. mtp53 silence enhanced PQR309-inhibited cell viability, spheroid formation, and the expressions of p-Akt, c-Myc, and CDK6. This is the first study to reveal the novel finding of the PI3K/mTOR dual inhibitor in lowering cell viability by abolishing the PI3K/Akt/mTOR/c-Myc/mtp53 positive feedback loop in endometrial cancer cell lines.

## 1. Introduction

Uterine corpus cancer is the most common gynecological malignancy in the world and in Taiwan. The Taiwan Health Promotion Administration of the Ministry of Health and Welfare reported that the age-standardized incidence rate of uterine corpus cancer was 12.99 per 100,000 women based on the annual cancer registry report in 2016. Most uterine corpus cancers are endometrial cancers, which originate from the uterine epithelium [1,2]. The incidence of endometrial cancer has been increasing in recent years. Based on the difference in tissue morphology, histology, and molecular profiles, endometrial cancer is traditionally divided into two types, type I and type II. More than 80% of endometrial cancers are common endometrioid endometrial cancer (EEC), which is classified as type I endometrial cancer [3]. In contrast, type II endometrial cancer includes serous endometrial cancer (SEC) and clear cell endometrial cancer (CCEC), which account for about 10% and 3%, respectively, as well as uterine carcinosarcoma (UCS), which is less than 2% [3]. Gene mutations in PIK3CA, PIK3R1, KRAS, PTEN, and PPP2R1A commonly detected in type I endometrial cancer cause high activation of the PI3K/Akt/mTOR pathway [4,5]. 

The PI3K/Akt/mTOR pathway is important in tumor development and metastasis [6]. Bimiralisib (PQR309), an orally bioavailable selective dual inhibitor of PI3K and mTOR, is still being studied in preclinical models and clinical trials [7]. PQR309 shows high specificity in kinase inhibition by CEREP BioPrint Panel [8]. PQR309 inhibits cell proliferation by inducing G1-phase cell cycle arrest in lymphoma cells and glioblastoma cells [9,10]. 

In this study, we explored the novel finding that the proliferation of endometrial cancer cells can be inhibited via the PI3K/Akt/mTOR/c-Myc/mtp53 positive feedback loop. We found that PQR309, a dual PI3K/mTOR inhibitor, reduces proliferation in endometrial cancer cells and endometrial cancer stem cells by decreasing CDK6 and increasing p27 and subsequently inducing G1-phase arrest. Inhibition of c-Myc/mtp53 cascade played a critical role in antiendometrial cancer by PQR309. This is the first study to reveal the novel function of the dual PI3K/mTOR inhibitor in decreasing cell viability by abolishing the PI3K/Akt/mTOR/c-Myc/mtp53 positive feedback loop in endometrial cancer cell lines.

## 2. Materials and Methods

### 2.1. Cells and Chemicals

AN3CA (ATCC, HTB-111) and HEC-1A (ATCC, HTB-112) cells were obtained from the American Type Culture Collection. HEC-59 (JCRB1120) and Ishikawa (JCRB1505) cells were purchased from the Japanese Collection of Research Bioresources (JCRB) cell bank. AN3CA cells were cultured and grown in Minimum Essential Medium (MEM) (GIBCO, 41500-034) supplemented with 10% fetal bovine serum (GIBCO, 10437). HEC-59 and Ishikawa cells were cultured in Minimum Essential Medium (MEM) (GIBCO, 41500-034) supplemented with 15% fetal bovine serum. HEC-1A cells were cultured in McCoy’s 5A Medium (SIGMA, M4892) supplemented with 10% fetal bovine serum. All cells were cultured at 37 °C in a humidified atmosphere of 5% CO_2._ PQR309 (Bimiralisib; 23441) and KJ-Pyr-9 (19116) were purchased from Cayman Chemical (Ann Arbor, MI). Chloroquine diphosphate salt (C 6628) was purchased from Sigma (St. Louis, MO, USA).

### 2.2. Cell Viability Assay

AN3CA (5 × 10^3^ cells/well), HEC-59 (6 × 10^3^ cells/well), Ishikawa (6 × 10^3^ cells/well), and HEC-1A (6 × 10^3^ cells/well) cells suspended in a 100 μL culture medium were seeded into a 96-well plate. Following treatment with PQR309 for 48 h, the treating medium was removed, and 100 μL of fresh medium containing 0.5 mg/mL 3-(4,5-dimethylthiazol-2-yl)-25-diphenyltetrazolium bromide (MTT; Sigma, M 2128) was added to the wells. MTT assay was performed as previously described [11]. 

### 2.3. Autophagosome Detection by CYTO-ID^®^ Autophagy Detection Kit 2.0

After treatment of PQR309 for 48 h, AN3CA and HEC-1A cells were stained with Cyto-ID Green Detection Reagent and Hoechst 33,342 Nuclear Stain (ENZ-KIT175-0200, Enzo Life Sciences, Farmingdale, NY, USA) for 20 min. The stained cells were investigated under a fluorescence microscope. 

### 2.4. Western Blot Assay

Anti-phospho-Akt Ser473 (#9271, Cell Signaling, Danvers, MA, USA), anti-Akt (#9272, Cell Signaling, Danvers, MA, USA), anti-LC3B (#3868, Cell Signaling, Danvers, MA, USA), anti-CDK6 (#13331, Cell Signaling, Danvers, MA, USA), anti-p27 (#sc1641, Santa Cruz Biotechnology, *Dallas*, TX, USA), anti-endoglin (AF1097, R&D, Minneapolis, MN, USA), anti-kallikrein 6 (KLK6; AF2008, R&D, Minneapolis, MN, USA), anti-p53 (M 7001, DakoCytomation, Glostrup, Denmark), anti-survivin (#2808, Cell Signaling, Danvers, MA, USA), anti-hTERT (#ab32020, abcam, Cambridge, UK), anti-c-Myc (MAB3696, R&D, Minneapolis, MN, USA), Anti-phospho-p70S6K Thr389 (#9234, Cell Signaling, Danvers, MA, USA), anti- p70S6K (#9202, Cell Signaling, Danvers, MA, USA), and anti-β-actin (AC-40, Sigma, St. Louis, MO, USA) were used to detect the expressions of indicated protein. The complete protocol for Western blot assay has been described in a previous publication [12].

### 2.5. 3 Dimension (3D) Tumor Spheroid Formation Assay

HEC-59, Ishikawa, and HEC-1A cells (1 × 10^3^ cells) were seeded into a well of an ultra-low-attachment 96-well plate (Corning Inc., Corning, NY, USA) containing 100 μL of culture medium. After incubation for 96 h, PQR309-containing medium was added to the well. The spheroids were incubated for another 7 days and observed under inverted light microscopy. The volume of the spheroids was calculated by the formula 0.5× larger diameter (mm) × small diameter (mm)^2^ [13].

### 2.6. Cancer Stem Cell Sphere Formation Assay

AN3CA cells (1 × 10^4^ cells) suspended in 2 mL sphere formation medium (DMEM/F12 medium containing 20 ng/mL epidermal growth factor (PeproTech Asia, Rehovot, Israel), 20 ng/mL basic fibroblast growth factor (PeproTech Asia, Rehovot, Israel), 4 μg/mL heparin (H3194, Sigma, St. Louis, MO, USA), 1 µg/mL Hydrocortisone (H0888, Sigma, St. Louis, MO, USA), 0.4% bovine serum albumin (15260037, Invitrogen, Paisley, UK), 5 μg/mL insulin (91077C, Sigma, St. Louis, MO, USA), and 1% methylcellulose (M0512, Sigma, St. Louis, MO, USA)) were seeded into a well of an ultra-low-attachment 6-well plate (Corning Inc., Corning, NY, USA). The cells were incubated for 10 days for sphere formation as the 1st sphere. Then, 2 × 10^4^ cells from the 1st were suspended in 2 mL sphere formation medium with or without PQR309 and seeded into a well of an ultra-low-attachment 6-well plate. A 500 μL fresh sphere formation medium was added to the well every 3 days. The cells were incubated for 14 days, and the 2nd spheres were observed via inverted light microscopy. The area and diameter of spheres were analyzed by ImageJ software (version 1.4.3.67).

### 2.7. Flow Cytometry

AN3CA, HEC-59, and Ishikawa cells were seeded into a 60 mm dish containing 4 mL of culture medium. After 16 h incubation, fresh medium with or without PQR309 was added to the dish. After treatment for 48 h, the cells were used to investigate apoptosis/cell death and cell cycle distribution. The Annexin V-FITC Apoptosis Detection Kit (556 547, BD Biosciences, San Jose, CA, USA) was used to investigate apoptosis and cell death. The fixed cells were stained with propidium iodine, and cell cycle distribution was analyzed by flow cytometry. The complete protocol for both analyses has been described elsewhere [14].

### 2.8. VSV-G Pseudotyped Lentivirus–shRNA Production and Infection

The shRNA expressing lentivirus was purchased from the National RNAi Core Facility located at the Institute of Molecular Biology/Genomic Research Center, Academia Sinica. Individual clones were identified by their unique TRC number: shLuc TRCN0000072249 for vector control targeted to luciferase; shp53 TRCN0000003755 (responding sequence: GTCCAGATGAAGCTCCCAGAA) targeted to p53. The detailed steps of lentivirus infection have been previously described [15].

### 2.9. Statistical Analysis

A one-sample *t*-test by Predictive Analytics SoftWare (PASW) Statistics 18 was used to conduct the statistical comparisons between two groups. Values of *p* < 0.05 were considered significant. Data are presented as mean ± SD.

## 3. Results

### 3.1. Dual PI3K/mTOR Inhibitor PQR309 Reduces Cell Viability in Endometrial Cancer Cell Lines

**Figure 1:**
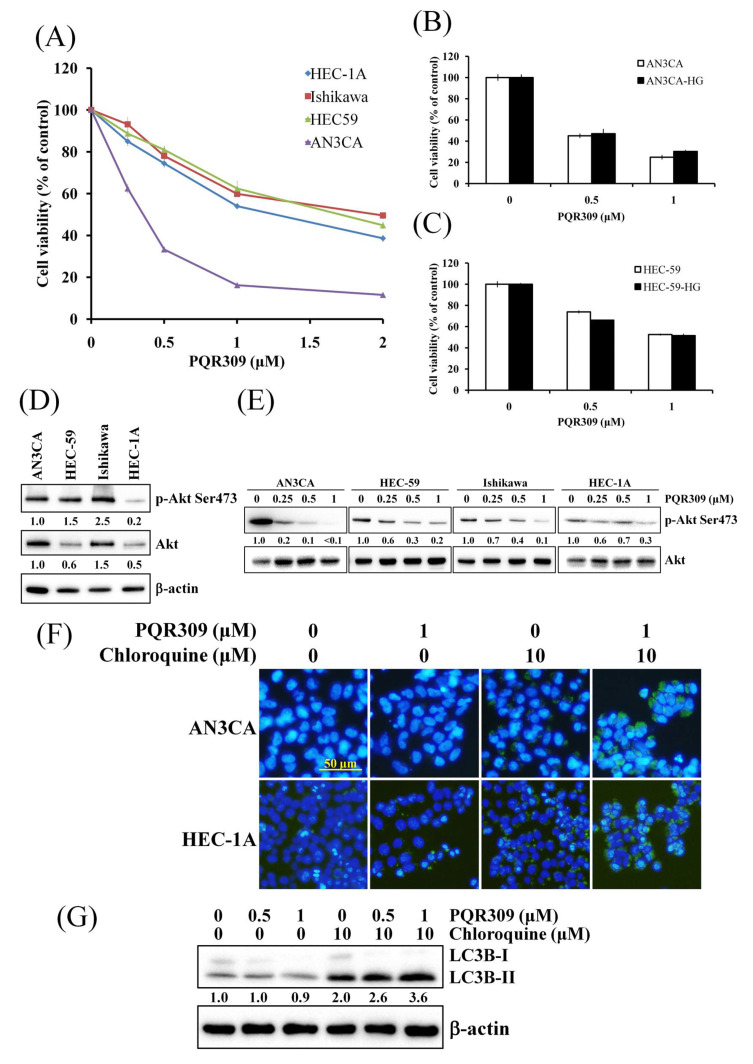
Effect of PQR309 on cell viability and autophagy induction in endometrial cancer cells. (**A**) AN3CA (5 × 10^3^ cells/well of 96-well plate), HEC-59 (6 × 10^3^ cells/well of 96-well plate), Ishikawa (6 × 10^3^ cells/well of 96-well plate), and HEC-1A (6 × 10^3^ cells/well of 96-well plate) were treated with various concentrations of PQR309 (0, 0.25, 0.5, 1, and 2 μM) for 72 h. Cell viability was analyzed by MTT assay. (**B**,**C**) After PQR309 (0, 0.5, and 1 μM) treatment for 72 h in indicated cells, cell viability was analyzed by MTT assay. HG, high-glucose medium culture. (**D**) Protein expressions of p-Akt, Akt, and β-actin were determined by Western blot. ImageJ software was used to quantify the band intensities of p-Akt and Akt. Data shown are the relative expression standardized by the β-actin protein level. The ratio of AN3CA was set at 1. (**E**) After PQR309 (0, 0.25, 0.5, and 1 μM) treatment for 48 h in indicated cells, equal amounts of total cell lysates were analyzed by Western blot assay. β-actin served as a loading control. ImageJ software was used to quantify the band intensities of p-Akt. Data shown are the relative expression standardized by the Akt protein level. The ratio of cells without treatment was set at 1. (**F**) After treatment of PQR309 for 48 h, AN3CA and HEC-1A cells were stained with the CYTO-ID Autophagy Detection Kit 2.0. The stained cells were investigated under a fluorescence microscope. Scale bar indicates 50 μm. (**G**) After PQR309 and chloroquine cotreatment for 48 h in AN3CA cells, LC3B-I and LC3B-II were analyzed by Western blot assay. β-actin served as a loading control. ImageJ software (version 1.4.3.67) was used to quantify the band intensities of LC3B-II. Data shown are the relative expression standardized by the β-actin protein level. The ratio of cells without treatment was set at 1.

Endometrial cancer cell lines, AN3CA, HEC-59, Ishikawa, and HEC-1A, were used to investigate the survival-inhibiting effect of PQR309 in endometrial cancer. As shown in Figure 1A, PQR309 inhibited cell viability of endometrial cancer cell lines in a dose-dependent manner. Furthermore, PQR309 suppressed the cell viability of primary endometrial cancer cells, EMC4 and EMC5 cells (Figure S1). Hyperglycemia is a risk factor of endometrial cancer and may alter the therapeutic effect of anticancer drugs [16]. AN3CA and HEC-59 cells were cultured in high-glucose medium for 2 weeks to adapt to high-glucose conditions (AN3CA-HG and HEC-59-HG cells). After PQR309 treatment for 48 h, no significant differences were investigated between the cells cultured in normal- and high-glucose medium, suggesting that high glucose does not alter the anticancer effect of PQR309 in endometrial cancer cells (Figure 1B,C). As shown in Figure 1D, different activities of Akt were investigated in different endometrial cancer cell lines. PQR309 markedly reduced the activation of Akt in endometrial cancer cells for 48 h (Figure 1E). The PI3K/Akt/mTOR pathway is important in autophagy induction [17]. To investigate autophagosome formation, the CYTO-ID Autophagy Detection Kit 2.0 was used to stain the cells after PQR309 treatment. PQR309 alone treatment did not induce autophagosome accumulation in endometrial cancer cells (Figure 1F). However, cotreatment with PQR309 and chloroquine, a lysosome inhibitor, induced higher autophagosome accumulation than chloroquine-alone treatment (Figure 1F). Furthermore, PQR309 and chloroquine cotreatment increased LC3B-II accumulation when compared to alone treatment of PQR309 or chloroquine, suggesting that PQR309 induces autophagic flux in endometrial cancer cell lines (Figure 1G).

### 3.2. Dual PI3K/mTOR Inhibitor PQR309 Inhibits Tumor Spheroid Formation of Endometrial Cancer Cell Lines

**Figure 2:**
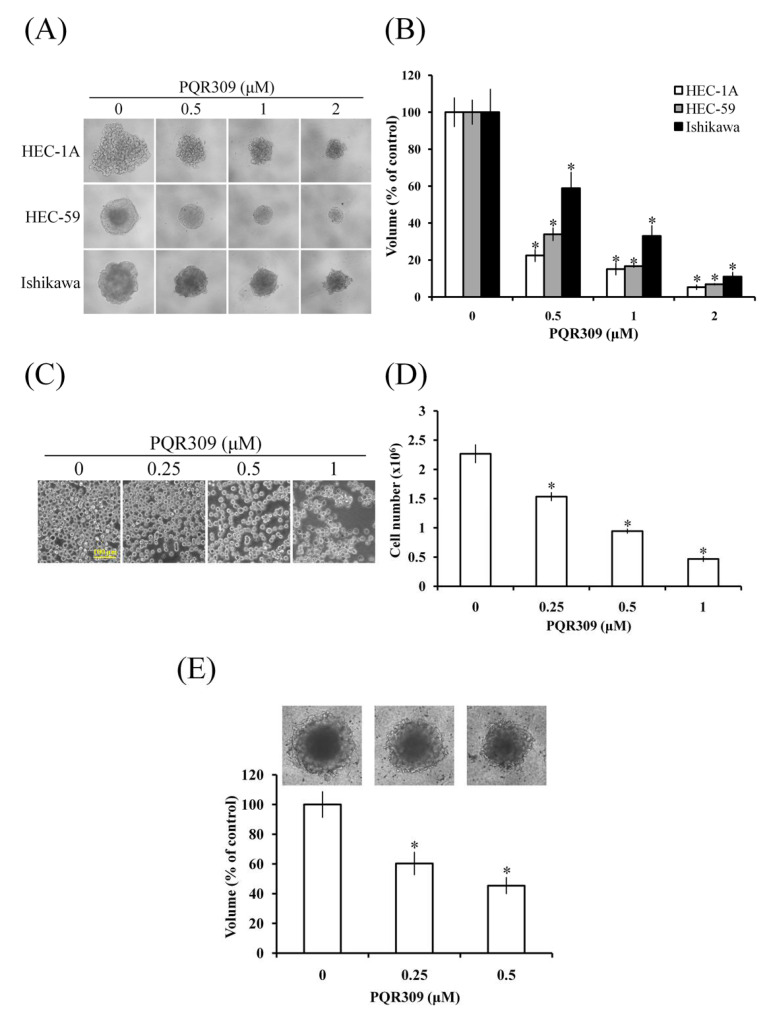
Effect of PQR309 on tumor spheroid formation in endometrial cancer cells. (**A**) HEC-1A, HEC-59, and Ishikawa cells (1 × 10^3^ cells/well of 96-well dish) were seeded onto ultra-low-attachment 96-well plates. After 96 h incubation, PQR309 (0, 0.5, 1, and 2 μM)-containing medium was added to the well, and the spheroids were incubated for 7 days. Spheroids were investigated under an inverted microscope. (**B**) The volumes of spheroids were determined by the formula 0.5 × larger diameter × small diameter^2^. Data show the relative spheroid volume, and the volume of spheroid without PQR309 treatment was set at 100%. (**C**) AN3CA-S cells (6 × 10^5^ cells/well of 6-well dish) were seeded onto ultra-low-attachment 6-well plates. Scale bar indicates 100 μm. (**D**) Cell number of (**C**) was counted under an inverted microscope. (**E**) HEC-59 cells (1 × 10^3^ cells/well of 96-well dish) were seeded onto ultra-low-attachment 96-well plates. After 96 h incubation, PQR309 (0, 0.25, and 0.5 μM)-containing medium with 25% Matrigel was added to the well, and the spheroids were incubated for 7 days. Spheroids were investigated under an inverted microscope. The volumes of spheroids of (**A**) were determined by the formula 0.5 × larger diameter × small diameter^2^. Data show the relative spheroid volume, and the volume of spheroid without PQR309 treatment was set at 100%. The symbol ∗ indicates *p* < 0.05.

The three-dimensional tumor spheroid assay is an efficient model to investigate drug effects in vitro. As shown in Figure 2A, HEC-1A, HEC-59, and Ishikawa cells formed spheroids in the ultra-low-attachment 96-well plate. PQR309 significantly reduced the size of tumor spheroids in a dose-dependent manner (Figure 2B). AN3CA cells were derived from the metastatic site and with antianoikis ability [18]. However, AN3CA cells did not aggregate and form tumor spheroids in the ultra-low-attachment 96-well plate (data not shown). We established that AN3CA-Suspension (AN3CA-S) cells can be cultured under suspension conditions. PQR309 reduced the cell number of AN3CA-S significantly (Figure 2C,D). The extracellular matrix (ECM) is important in tumor development and growth [19]. Tumor spheroid formation in Matrigel was performed to investigate the inhibiting effect of PQR309 with ECM. As shown in Figure 2E, PQR309 significantly suppressed the growth of tumor spheroids in ECM.

### 3.3. Dual PI3K/mTOR Inhibitor PQR309 Inhibits Cancer Stem Cell Sphere Growth of Endometrial Cancer Cell Lines

**Figure 3:**
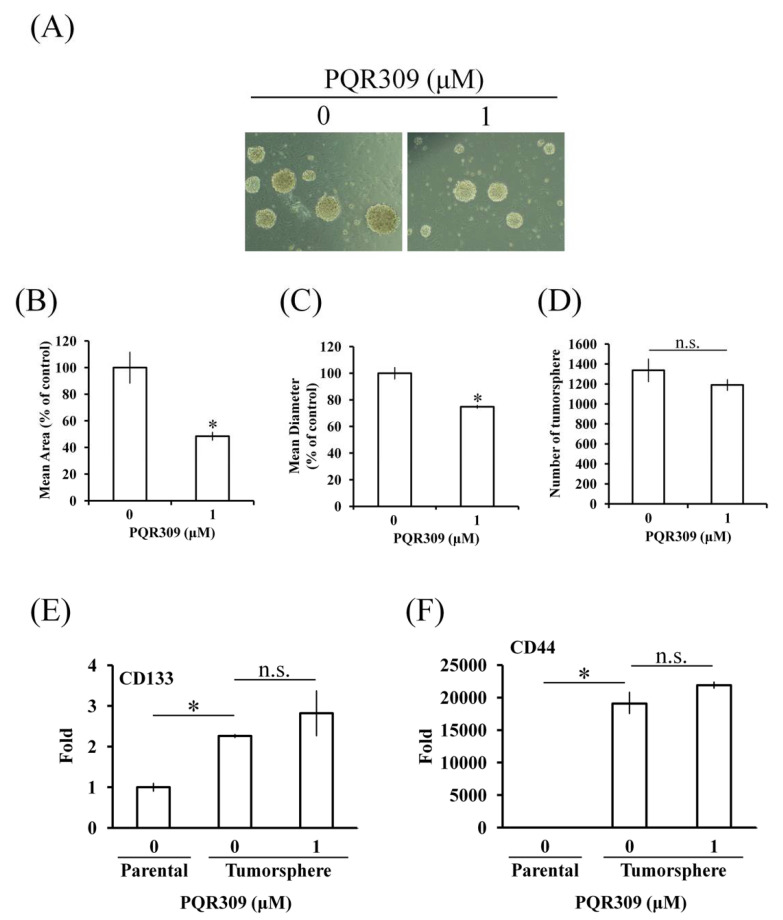
Effect of PQR309 on tumorsphere formation in AN3CA cells. (**A**) Cancer stem cells of AN3CA (2 × 10^4^ cells/well of a 6-well plate) from the first tumorsphere were seeded in the sphere formation medium with or without 1 μM PQR309. After 14 days, the spheres were investigated under an inverted microscope. (**B**,**C**) The area and diameter of spheres were analyzed by Software ImageJ. (**D**) The spheres were counted under an inverted microscope. Total RNA was collected from spheres after treating with PQR309 (0 and 1 μM) for 14 days. RT-qPCR was performed to analyze the mRNA expressions of (**E**) CD133 and (**F**) CD44. The ratio of parental AN3CA cells without treatment was set at 1. The symbol ∗ indicates *p* < 0.05. “n.s.” indicates no significance (*p* > 0.05).

Cancer stem cells play an important role in tumor development, chemotherapy resistance, cancer recurrence, and metastasis [20]. Cancer stem cell sphere assay was performed to investigate the effect of PQR309 on the cancer stem cells of AN3CA cells. After PQR309 treatment, the size of the cancer stem cell sphere was reduced significantly (Figure 3A–C). However, the number of tumorspheres was not altered by PQR309 (Figure 3D). To further investigate the stemness alteration of tumorspheres, RT-qPCR was performed to investigate the mRNA expression of stem cell markers, CD133 and CD44. The mRNA expressions of CD133 and CD44 were upregulated in tumorspheres when compared to parental cells (Figure 3E,F). However, PQR309 did not alter the expressions of CD133 and CD44 in tumorspheres (Figure 3E,F). These results demonstrate that PQR309 inhibits the growth of cancer stem cells but does not reduce stemness.

### 3.4. Dual PI3K/mTOR Inhibitor PQR309 Induces Cell Cycle Arrest in Endometrial Cancer Cell Lines

**Figure 4:**
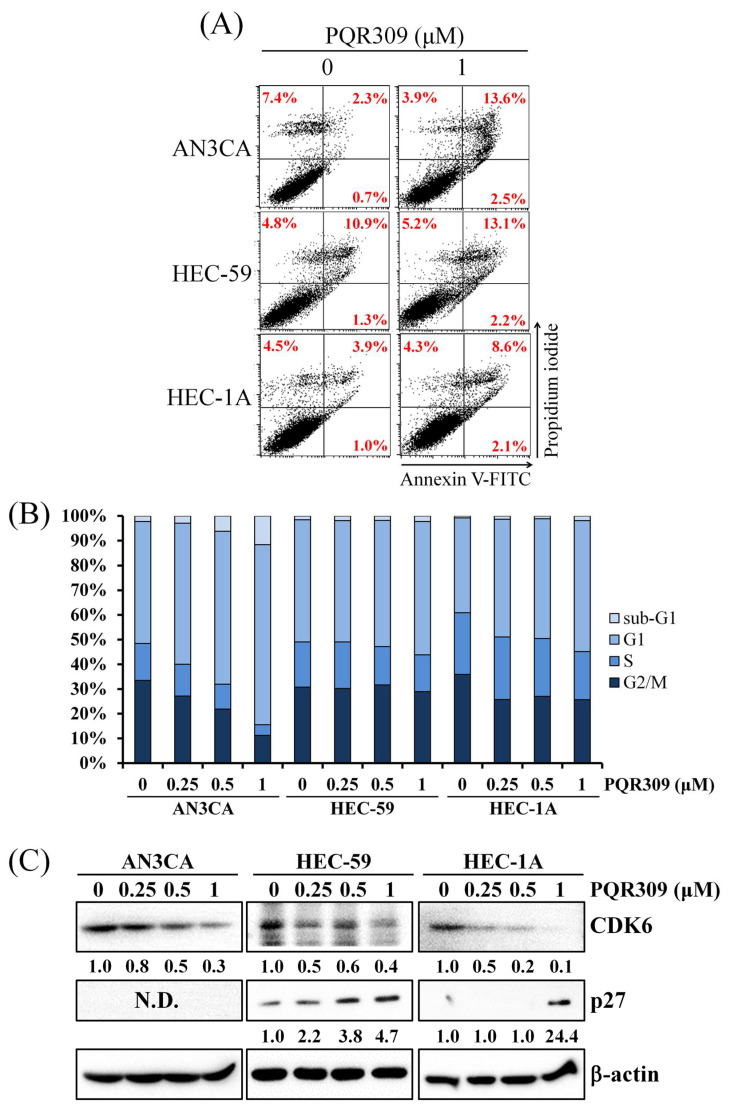
Effect of PQR309 on cell death and cell cycle arrest in endometrial cancer cells. (**A**) After treatment with PQR309 (0 and 1 μM) for 48 h, indicated endometrial cancer cells were stained with annexin V-FITC/propidium iodine and analyzed by flow cytometry. (**B**) AN3CA, HEC-59, and HEC-1A cells were treated with PQR309 (0, 0.25, 0.5, and 1 μM) for 48 h. After PI staining, cells were analyzed by flow cytometry. (**C**) After treating with PQR309 in AN3CA, HEC-59, and HEC-1A cells for 48 h, protein expressions of CDK6 and p27 were determined by Western blot. ImageJ software was used to quantify the band intensities of CDK6 and p27. Data shown are the relative expression standardized by the β-actin protein level. The ratio of cells without treatment was set at 1. N.D., not detected.

To evaluate the mechanism of cell viability inhibition by PQR309, annexin V/propidium iodide staining assay was used to analyze cell death. As shown in Figure 4A, PQR309 slightly induced apoptosis and cell death in endometrial cancer cells. Cell cycle distribution was analyzed by flow cytometry and indicated that PQR309 causes an increase in the G1 population and a decrease in the S population in endometrial cancer cells (Figure 4B). PQR309 decreased the protein expression of CDK6 in endometrial cancer cells (Figure 4C). Furthermore, induction of p27 by PQR309 was observed in HEC-59 and HEC-1A cells (Figure 4C). These results demonstrate that PQR309 inhibits the cell viability of endometrial cancer cells by eliciting G1-phase cell cycle arrest. 

### 3.5. Dual PI3K/mTOR Inhibitor PQR309 Inhibits Mutant p53 and c-Myc in Endometrial Cancer Cell Lines

**Figure 5:**
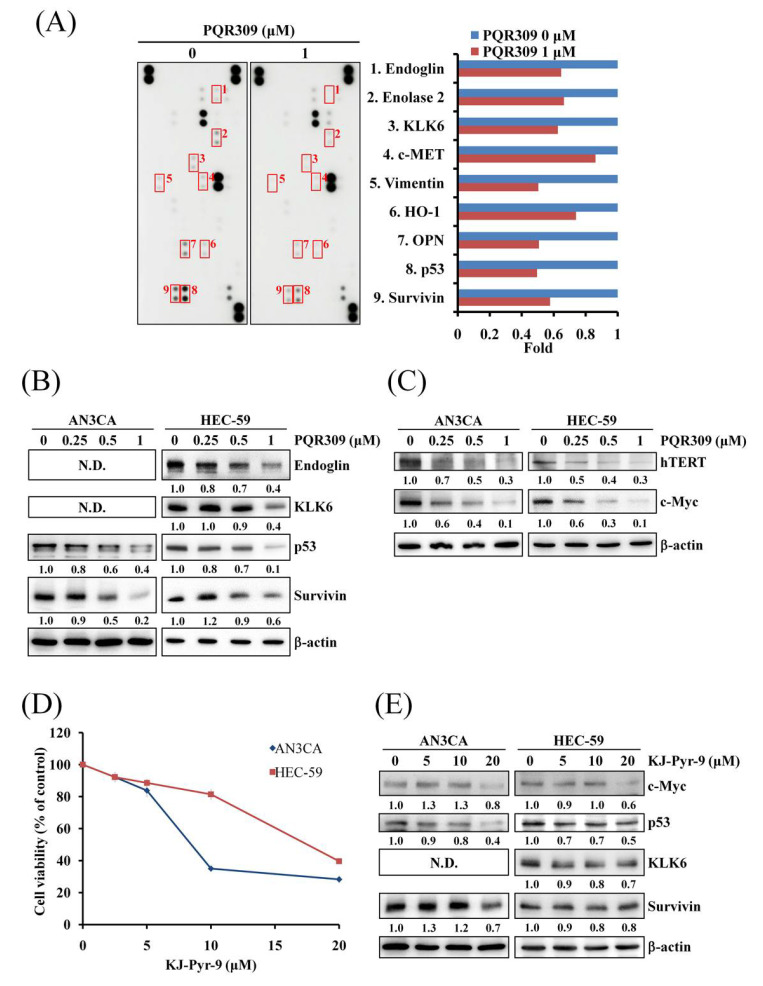
Effect of PQR309 on the expression of c-Myc and its possible target genes. (**A**) After treating with PQR309 for 48 h in HEC-59 cells, Proteome Profiler Human XL Oncology Array was performed according to the manufacturer protocol. The dot intensities were quantified by ImageJ software. The ratio of cells without treatment was set at 1. (**B**) After treating with PQR309 in AN3CA and HEC-59 cells for 48 h, protein expressions of endoglin, KLK6, p53, and survivin were determined by Western blot. ImageJ software was used to quantify the band intensities of endoglin, KLK6, p53, and survivin. Data shown are the relative expression standardized by the β-actin protein level. The ratio of cells without treatment was set at 1. (**C**) Western blot assay was performed to detect the expressions of hTERT and c-Myc in AN3CA and HEC-59 cells after PQR309 treatment for 48 h. ImageJ software was used to quantify the band intensities of hTERT and c-Myc. Data shown are the relative expression standardized by the β-actin protein level. The ratio of cells without treatment was set at 1. (**D**) AN3CA (5 × 10^3^ cells/well of 96-well plate) and HEC-59 (6 × 10^3^ cells/well of 96-well plate) were treated with KJ-Pyr-9 (0, 2.5, 5, 10 and 20 μM), an inhibitor of c-Myc, for 72 h. Cell viability was analyzed by MTT assay. (**E**) Protein expressions of c-Myc, p53, KLK6, and survivin were determined by Western blot. ImageJ software was used to quantify the band intensities of c-Myc, p53, KLK6, and survivin. Data shown are the relative expression standardized by the β-actin protein level. The ratio of cells without treatment was set at 1. N.D., not detected.

To further investigate the mechanism of the anticancer effect of PQR309 on endometrial cancer cells, the Proteome Profiler Human XL Oncology Array was performed to detect differences in 84 cancer-related proteins. As shown in Figure 5A, when compared to the control, PQR309 altered the expression of nine proteins in HEC-59 cells, including endoglin, enolase 2, kallikrein-6 (KLK6), c-MET, vimentin, *Heme oxygenase*-1 (HO-1), *Osteopontin* (OPN), p53, and survivin (Figure 5A). Western blot assay was performed to check the expressions of the proteins mentioned above. As shown in Figure 5B, PQR309 decreased the expressions of endoglin, KLK6, p53, and survivin. Protein expressions of endoglin and KLK6 could not be detected in AN3CA cells (Figure 5B). HO-1 was not detected in both cells (data not shown). Furthermore, according to the ENCODE Transcription Factor Targets dataset on Harmonizome and paper searching, we found that KLK6, p53, and survivin are target genes of c-Myc [21,22,23]. Therefore, the protein expression of c-Myc was analyzed. As shown in Figure 5C, PQR309 inhibited c-Myc expression obviously. PQR309 also decreased the expression of hTERT, a well-known target gene of c-Myc. KJ-Pyr-9, a c-Myc inhibitor inhibiting c-Myc activity by disrupting the interaction of Myc and Max, was used to investigate the effect of c-Myc inhibition on endometrial cancer cells. KJ-Pyr-9 inhibited cell viability in AN3CA and HEC-59 (Figure 5D). KJ-Pyr-9 decreased the expression of p53, KLK6, survivin, and hTERT (Figure 5E and Figure S2).

### 3.6. p53 Knockdown Decreases Cell Proliferation and Akt/mTOR Activity

**Figure 6:**
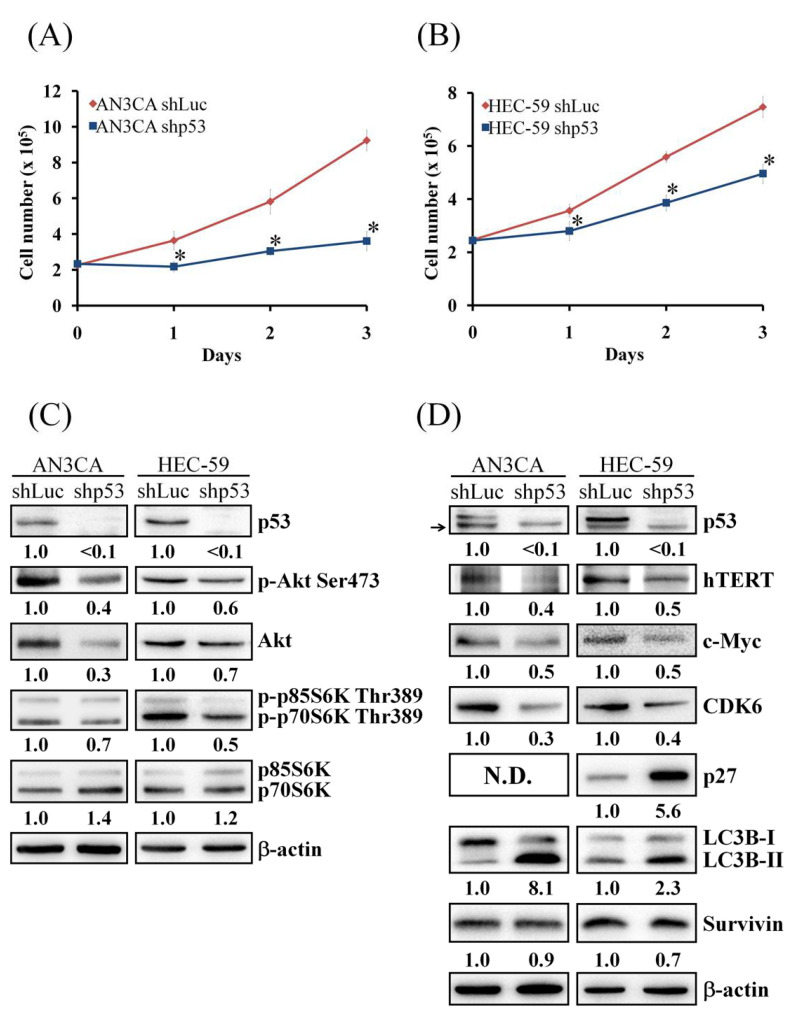
Effect of mtp53 knockdown on cell proliferation and Akt/mTOR pathway. (**A**) AN3CA shLuc and shp53 cells (2 × 10^5^ cells/35 mm dish) were seeded on a 35 mm dish. After 24 (Day 0), 48 (Day 1), 72 (Day 2), and 96 h (Day 3) incubation, the cells were harvested by trypsin and counted under an inverted microscope. (**B**) HEC-59 shLuc and shp53 cells (2.5 × 10^5^ cells/35 mm dish) were seeded on a 35 mm dish. After 24 (Day 0), 48 (Day 1), 72 (Day 2), and 96 h (Day 3) incubation, the cells were harvested by trypsin and counted under an inverted microscope. (**C**) Protein expressions of p53, p-Akt, Akt, p- p-p85S6K/p70S6K, and p85S6K/p70S6K were determined by Western blot. ImageJ software was used to quantify the band intensities of p53, p-Akt, Akt, p-p85S6K/p70S6K, and p85S6K/p70S6K. Data shown are the relative expression standardized by the β-actin protein level. The ratio of shLuc cells was set at 1. (**D**) Protein expressions of p53, hTERT, c-Myc, CDK6, p27, LC3B, and survivin were determined by Western blot. The arrow indicated the nonspecific band. ImageJ software was used to quantify the band intensities of indicated protein. Data shown are the relative expression standardized by the β-actin protein level. The ratio of shLuc cells was set at 1. The symbol ∗ indicates *p* < 0.05. N.D., not detected.

Mutation of p53 is important in the development of endometrial cancer and could be found in most endometrial cancer cell lines [24,25]. All endometrial cancer cell lines used in this study harbor mutant p53 (mtp53). In Figure 5, we found that PQR309 inhibits c-Myc and its target genes. KJ-Pyr-9, an inhibitor of c-Myc, inhibits cell viability in AN3CA and HEC-59 cells similarly to PQR309. Furthermore, the inhibiting effect of KJ-Pyr-9 on p53 is better than KLK6 and survivin. These results demonstrate that PQR309 inhibits endometrial cancer cells by suppressing the c-Myc/mtp53 cascade. To elucidate the role of mtp53 in endometrial cancer cells, p53-targeting shRNA was used to inhibit the expression of p53. p53 silencing significantly lowered the proliferation in AN3CA and HEC-59 cells (Figure 6A,B). p53 silencing decreased the phosphorylation of Akt and p70S6K (Figure 6C). The protein expressions of hTERT, c-Myc, and CDK6 were decreased in AN3CA and HEC-59 cells (Figure 6D). p27 and LC3B-II were increased after silencing of p53 (Figure 6D). p53 silencing slightly inhibited the expression of survivin (Figure 6D). These results demonstrate that mtp53 promotes endometrial cancer cells growth as an activator of the Akt/mTOR pathway.

### 3.7. p53 Knockdown Enhances PQR309-Inhibited Cell Viability

**Figure 7:**
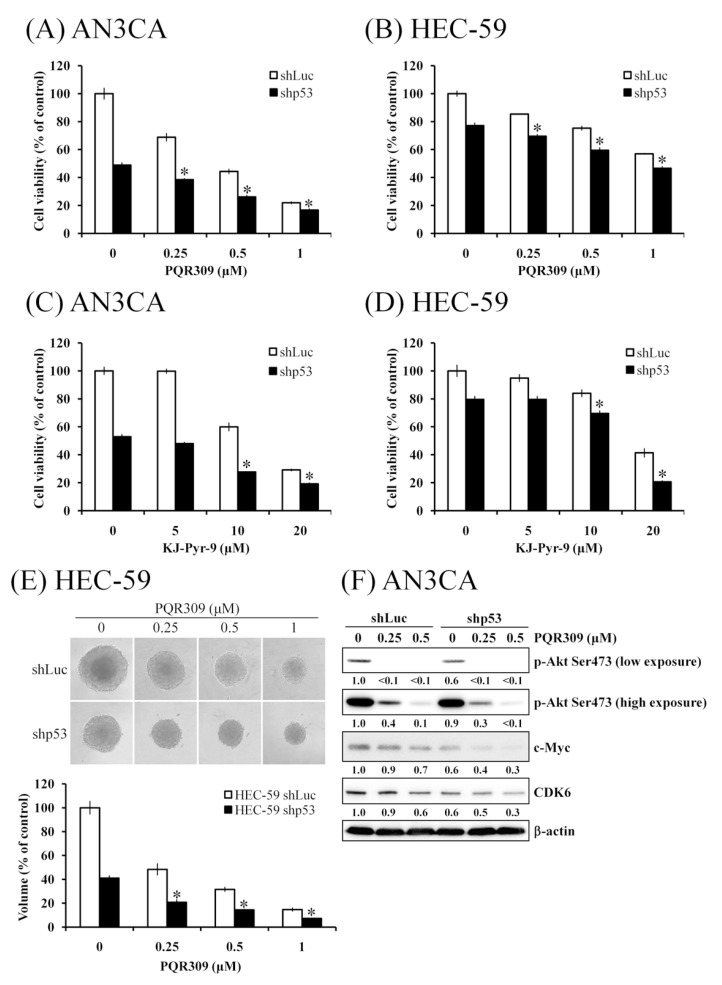
Effect of mtp53 knockdown on PQR309-mediated cell viability and tumor spheroid formation (**A**,**B**) AN3CA shLuc (5 × 10^3^ cells/well of 96-well plate), AN3CA shp53 (5 × 10^3^ cells/well of 96-well plate) and HEC-59 shLuc (6 × 10^3^ cells/well of 96-well plate), HEC-59 shp53 (6 × 10^3^ cells/well of 96-well plate) were treated with PQR309 (0, 0.25, 0.5 and 1 μM) for 72 h. Cell viability was analyzed by MTT assay. (**C**,**D**) AN3CA shLuc (5 × 10^3^ cells/well of 96-well plate), AN3CA shp53 (5 × 10^3^ cells/well of 96-well plate) and HEC-59 shLuc (6 × 10^3^ cells/well of 96-well plate), HEC-59 shp53 (6 × 10^3^ cells/well of 96-well plate) were treated with KJ-Pyr-9 (0, 5, 10 and 20 μM) for 72 h. Cell viability was analyzed by MTT assay. (**E**) HEC-59 shLuc and shp53 cells (1 × 10^3^ cells/well of 96-well dish) were seeded onto ultra-low-attachment 96-well plates. After 96 h incubation for spheroid formation, PQR309 (0, 0.25, 0.5, and 1 μM)-containing medium was added to the well, and the spheroids were incubated for 7 days. Spheroids were investigated under an inverted microscope. The volumes of spheroids were determined by the formula 0.5 × larger diameter × small diameter2. Data show the relative spheroid volume, and the volume of spheroids of HEC-59 shLuc without PQR309 treatment was set at 100%. (**F**) Protein expressions of p-Akt, c-Myc, and CDK6 were determined by Western blot. ImageJ software was used to quantify the band intensities of p-Akt, c-Myc, and CDK6. Data shown are the relative expression standardized by the β-actin protein level. The ratio of shLuc cells without treatment was set at 1. The symbol ∗ indicates *p*  <  0.05 for the PQR309-treated shp53 group when compared with the PQR309 or shp53 alone group.

In Figure 6, we found that mtp53 is an activator of the Akt/mTOR pathway in endometrial cancer cells. Further investigation of the effect of mtp53 on PQR309 was performed. Knockdown of mtp53 enhanced the inhibiting effect of PQR309 on cell viability in AN3CA and HEC-59 cells (Figure 7A,B). Cell viability suppression by KJ-Pyr-9, an inhibitor of c-Myc, also enhanced in shp53 cells when compared to shLuc (Figure 7C,D). The size of tumor spheroid was much lower in HEC-59 shp53 cells when compared to HEC-59 shLuc with or without PQR309 treatment (Figure 7E). The inhibiting effect of PQR309 on p-Akt, c-Myc, and CDK6 was enhanced in HEC-59 shp53 cells when compared to HEC-59 shLuc (Figure 7F). These results demonstrated that PQR309 inhibits cell viability in endometrial cancer cells by abolishing the positive feedback loop of Akt/mTOR/c-Myc/mtp53.

## 4. Discussion

PTEN is the most frequently mutated gene in endometrial cancer. Mutations in *PIK3CA*, *PIK3R1*, and *KRAS* also occur at high frequency in endometrial carcinoma [4]. Most endometrial cancer cell lines have one or more mutations and/or copy number alterations in the *PIK3CA*, *PTEN*, and *KRAS* genes [26,27]. These mutations lead to high activation of the PI3K/Akt/mTOR pathway in endometrial cancer. Kim et al. generated *Pten* loss and *K-ras* mutation in progesterone receptor-positive cells in mice to study the development of endometrial cancer [28]. In their study, the expression of p-Akt is much higher in mice with *Pten* ablation when compared to mice with *K-ras* mutation [28]. Furthermore, the expression of p-Akt in mice with *Pten/K-ras* double mutation is not higher than mice with *Pten* loss. PTEN is mutated, and the expression of PTEN is lost in AN3CA, HEC-59, and Ishikawa cells [26]. HEC-1A cells possess mutation in KRAS but not in PTEN. In Figure 1D, the result shows that the expression of p-Akt is higher in AN3CA, HEC-59, and Ishikawa when compared to HEC-1A. Our result was similar to the finding of Kim et al. Interestingly, coexistence of PTEN mutation and KRAS mutation in the endometrial cancer cell line is very rare, suggesting that PTEN mutation and KRAS mutation may be driving mutations of tumor development. 

The most common mutation in type II endometrial cancer, SEC, CCEC, and UCS, is p53 mutation [4,5]. The second most common mutation gene is protein phosphatase 2 regulatory subunit A, alpha (PPP2R1A), and the protein expressed by PPP2R1A is one of the subunits of protein phosphatase 2A (PP2A) [29]. It is known that PP2A is a tumor suppressor gene and has the ability to inhibit the PI3K/Akt/mTOR pathway. Mutation of PPP2R1A destroys the function of PP2A and highly activates the PI3K/Akt/mTOR pathway [30,31]. The inhibiting effect of PI3K/Akt/mTOR/c-Myc/mtp53 by PQR309 may also work in type II endometrial cancer.

The PI3K/Akt/mTOR signaling pathway is the key regulator of autophagy [32]. Autophagosomes and LC3-II are well-known markers of autophagy induction [17]. In Figure 1F,G, PQR309 alone did not induce accumulation of autophagosomes and LC3B-II. However, the expression of autophagosomes and LC3B-II is higher in cells after cotreatment with PQR309 and chloroquine when compared to chloroquine alone. These results demonstrate that PQR309 elicits a balanced autophagic flux in endometrial cancer cells. 

It has been reported that cancer stem cells play an important role in tumor growth and progression [33]. Several signaling pathways participate in cancer stem cell proliferation and survival, including the PI3K/Akt/mTOR pathway [34]. CD133 and CD44 are well-known markers of cancer stem cells [35]. Beyond being markers, CD133 and CD44 can activate the PI3K/Akt pathway to drive tumor-initiating cells [36,37,38]. Interaction of CD44 and its ligand, hyaluronan, activates the Src/ERK signaling pathway, leading to acquired resistance to PI3Kα inhibitor, BLY719, in luminal breast carcinomas [39]. In the present study, CD133 and CD44, especially CD44, were upregulated in endometrial cancer cells in tumorsphere formation assay, suggesting that stemness is increased in this cancer stem cell model (Figure 3E,F). We found that dual PI3K/mTOR inhibitor PQR309 lowers the size of the sphere but does not alter the number of spheres (Figure 3B–D). Furthermore, PQR309 did not decrease the expressions of CD133 and CD44 in the tumorsphere. On the contrary, an increasing trend of expressions of CD133 and CD44 in the PQR309-treating tumorsphere could be investigated, suggesting that a rescue effect was triggered. Taken together, these results suggest that PQR309 inhibits the proliferation of endometrial cancer stem cells but does not lower stemness. 

c-Myc, a well-known oncogene, regulates expressions of several cancer-related genes [40]. According to the Human Protein Atlas, high expression of c-Myc leads to a lower survival probability of patients with endometrial cancer. Upregulation of c-Myc induces growth, epithelial–mesenchymal transition, and drug resistance in endometrial cancer cells [41]. c-Myc amplification and activation can be a resistant mechanism of inhibitors of the PI3K/Akt/mTOR pathway [42,43]. In this study, c-Myc and its target genes were inhibited by PQR309 (Figure 5B,C). KJ-Pyr-9, an inhibitor of c-Myc, decreased the cell viability of PQR309 in AN3CA and HEC-59 cells (Figure 5D). However, the inhibiting effects of KJ-Pyr-9 on KLK6 and survivin were lower than p53 and hTERT (Figure 5E and Figure S2). This result suggested that besides c-Myc, other transcription factors regulate the expressions of KLK6 and survivin in endometrial cancer.

mtp53 is important in the development and progress of several types of tumors, including endometrial cancer [44]. Not only loss of the anticancer ability of wtp53 but also gain-of-function of mtp53 promotes tumor growth and metastasis [44]. mtp53 activates the PI3K/Akt signaling pathway by interfering in the interaction of DAB2IP with PI3K and Akt [45,46]. In this study, PQR309 inhibited mtp53 expression in endometrial cancer cells by inhibiting c-Myc (Figure 5B). Silencing of mtp53 led to inhibition of cell viability and the Akt/mTOR pathway in endometrial cancer cells (Figure 6C). Therefore, the effect of mtp53 silencing was similar to PQR309 in endometrial cancer cells (Figure 6D). Furthermore, mtp53 silencing enhanced PQR309-mediated inhibition of cell viability and expressions of p-Akt, c-Myc, and CDK6 (Figure 7). Taken together, mtp53 activated the PI3K/Akt/mTOR pathway in endometrial cancer.

## 5. Conclusions

**Figure 8:**
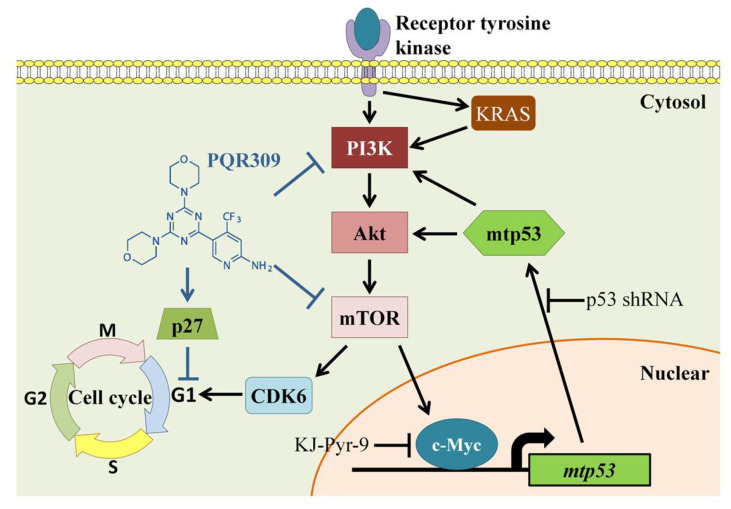
A suggested model of PQR309 induced cell cycle arrest by inhibiting PI3K/Akt/mTOR/c-Myc/mtp53 positive feedback loop in endometrial cancer cells.

In conclusion, the dual PI3K/mTOR inhibitor PQR309 inhibits cell viability in two-dimensional (2D) and three-dimensional (3D) cell culture models in endometrial cancer cells. It also decreases the proliferation of endometrial cancer stem cells. The dual inhibitor blocks the positive feedback loop of PI3K/Akt/mTOR/c-Myc/mtp53, leading to G1-phase arrest of the cell cycle (Figure 8). This study explores new insights into the possible regulatory effect of the dual PI3K/mTOR inhibitor such as PQR309 on the positive feedback loop of PI3K/Akt/mTOR/c-Myc/mtp53 and the role of this loop in endometrial cancer cell proliferation. This study provides evidence of PQR309 on endometrial cancer inhibition, and it may be a potential drug in antiendometrial cancer.

## Data Availability

Data from the manuscript are available from the corresponding author.

## Supplementary Materials

The following are available online at https://www.mdpi.com/article/10.3390/cells10112916/s1, Figure S1: Effect of PQR309 on cell viability of primary endometrial cancer cells. EMC4 and EMC5 cells (5 × 10^3^ cells/well of 96-well plate) were treated with various concentrations of PQR309 (0, 0.25, 0.5, 1, and 2 μM) for 72 h. Cell viability was analyzed by MTT assay, Figure S2: Effect of KJ-Pyr-9, an inhibitor of c-Myc, on hTERT expression in AN3CA cells Western blot assay was performed to detect the expressions of hTERT in AN3CA cells after KJ-Pyr-9 treatment for 48 h. ImageJ software was used to quantify the band intensities of hTERT. Data shown are the relative expression standardized by the β-actin protein level. The ratio of cells without treatment was set at 1.
