# Supplementary material for: Suppression of PI3K/Akt/mTOR/c-Myc/mtp53 Positive Feedback Loop Induces Cell Cycle Arrest by Dual PI3K/mTOR Inhibitor PQR309 in Endometrial Cancer Cell Lines
Source: Cells. 2021 Oct 27;10(11):2916. doi: 10.3390/cells10112916 (PMC8616154; doi:10.3390/cells10112916)
Supplement: Supplementary file 1 [file cells-10-02916-s001.zip › cells-1401956-supplementary.pdf]

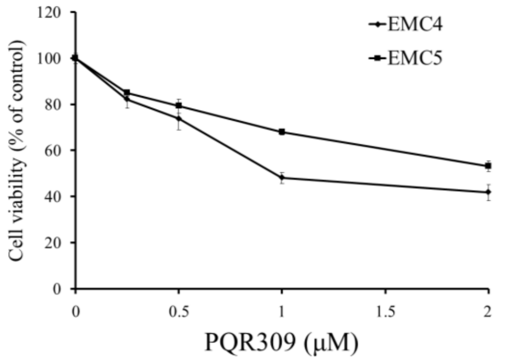

Supplementary Figure S1. Effect of PQR309 on cell viability of primary endometrial cancer cells

EMC4 and EMC5 cells (5 × 10^3 cells/well of 96-well plate) were treated with various concentrations of PQR309 (0, 0.25, 0.5, 1 and 2 μM) for 72 h. Cell viability was analyzed by MTT assay.

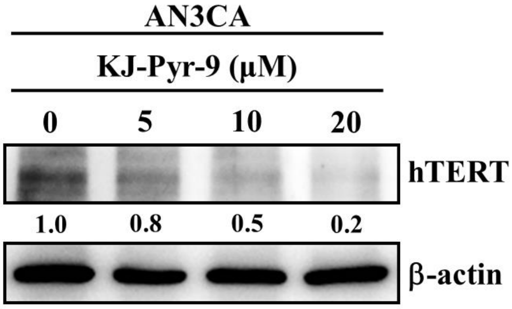

Supplementary Figure S2. Effect of KJ-Pyr-9 on hTERT expression in AN3CA cells

Western blot assay was performed to detect the expressions of hTERT in AN3CA cells after KJ-Pyr-9 treatment for 48h. Software ImageJ was used to quantify the band intensities of hTERT. Data shown are the relative expression standardized by the β-actin protein level. The ratio of cells without treatment was set at 1.
